# “Annual Pharma Statistics” as a Health Information System Application in Iranian Pharmaceutical Sector

**Published:** 2015

**Authors:** Amir Farshchi, Abbas Kebriaeezadeh, Rassoul Dinarvand


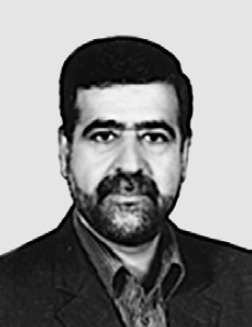


**Figure 1:**
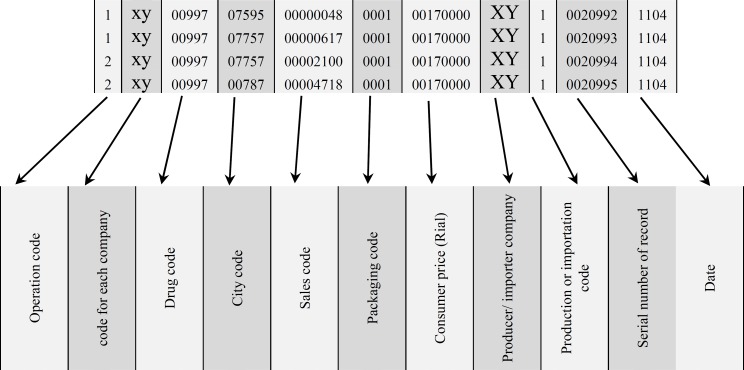
Sample format of MOH Annual Pharma Statistics data.

Information plays a vital role in effective management of any system. With the Health Management Information System (HMIS), specific information may be provided to support the decision makers at various levels of the health system to assist evidence-based decision making. It is almost three decades (from 1984) that Iranian Ministry of Health (MoH) has used HMIS in its pharmaceutical sector as a mean to have an efficient regulation. Pharmaceutical distributor companies have reported their sales data to MoH at monthly intervals. Each company should report its data by 5th of each month. “Iranian MoH Annual Pharma Statistics” or “Amarnameh daruee Iran” will be published as software after combining this data with other related data and statistical analysis. This database is a basis for deciding issues of many policy makers, and management possesses in pharmaceutical sector. After an efficient coordination between MoH and distributors, each company reports its data in a defined format including some detailed information. Table 1 shows a sample chart of characteristics for this HMIS. Each pharmaceutical product in each city/each month has one record. For each product with brand name, the specific code will be used. If there is not any code for branded products, it is very important to get feedback to MoH to solve such problems. Figure 1 is a sample file format of a hypothetical company named XY to show more details. Until 1998 this information was reported as a manual printed database. From 1998 to 2003 it was reported as a book, and since 2003 the electronic database including data since 1998 has been published. 

**Table 1:** Characteristics of MOH Annual Pharma Statistics variables

**Variable**	**Number of each variable character**	**Definition of each character**	**Number of lines for each variable**
Operation code	One digit	This code is for determining that each data in its line is for witch condition? For example: Sales (Code 1), Stock (Code 2), Rejects from sales (Code 3)	1
Two letter code for each company	Two digits	This code is special for each company	2-3
Drug code	Five digits	-	4-8
City code	Five digits	-	9-13
Sales (Number of boxes) in each month	Eight digits	-	14-21
Packaging (number of units in each box)	Four digits	-	22-25
Consumer price (Rial)	Eight digits	For each box	26-33
Three letter code for producer company	Three digits	This code is for producer or importer company	34-36
Production or importation code	One digits	1: Production 2: importation	37
Serial number of record	Seven digits	For each records from company	38-44
Date	Four digits	yymm	45-48

The growing use of high-throughput technologies in health management and the complexity of data generated in such area have created a need for dedicated software systems in order to collect, store and manage the data. We know such HMISs play an important role in healthcare management; however, more efforts should be done to upgrade the system based on new demands. For example; special attention to benchmark from other countries and adopting this system for the pharmaceutical industry will improve this HMIS. Furthermore, such HMISs could be designed and implemented for providing updated and relevant information on the consumption, and rational drug use for policy makers. To justify this purpose, we must always endeavor to have a dynamic system to collect and evaluate the relevant information. “Annual Pharma Statistics” is a good example for implementing health information system in pharmaceutical sector in Iran.

*Abbas Kebriaeezadeh is currently working as Professor of Department of Pharmacoeconomics and Pharmaceutical Administration, School of Pharmacy, Tehran University of Medical Science, Tehran, Iran. He could be reached at the following e-mail address:* kebriaee@tums.ac.ir

